# Krukenberg tumour as the initial manifestation of lung adenocarcinoma

**DOI:** 10.1002/rcr2.1133

**Published:** 2023-04-04

**Authors:** Guat Yee Lim, Yen Shen Wong, Zahrah Tawil, Roqiah Fatmawati Abdul Kadir, Annamalai Ramanathan, Aisya Natasya Musa

**Affiliations:** ^1^ Department of Internal Medicine Hospital Selayang Batu Caves Malaysia; ^2^ Faculty of Medicine University Teknologi MARA (UiTM) Sg Buloh Selangor Malaysia; ^3^ Department of Pathology Hospital Selayang Batu Caves Malaysia; ^4^ Department of Radiology University Teknologi MARA (UiTM) Sg Buloh Selangor Malaysia; ^5^ Department of Obstetrics & Gynecology Hospital Selayang Batu Caves Malaysia

**Keywords:** Krukenberg tumour, lung adenocarcinoma, ovarian tumour, pleural effusion

## Abstract

Krukenberg tumours are unusual metastatic tumours of the ovary with primary tumours from the stomach, breast and gastrointestinal malignancies. Krukenberg tumour from pulmonary malignancy represents an extremely rare situation. This is an elaboration of a case of young women with Krukenberg tumour rising from lung adenocarcinoma. A 38‐year‐old woman presented with progressive abdominal distention for the past 2‐years. Computed tomography (CT) of thorax, abdomen and pelvis revealed a huge ovarian mass with left lung nodules and left‐sided pleural effusion. A detailed immunohistochemical staining on pleural fluid cytology confirmed the diagnosis of metastatic adenocarcinoma of lung origin. She underwent doublet platinum chemotherapy as molecular testing for oncogenic mutation was negative. The patient responded well to chemotherapy with a significant reduction in ovarian tumour size. Early identification of the primary source of Krukenberg tumour is paramount to avoid invasive diagnostic surgical intervention for ovarian metastasis.

## Graphical abstract

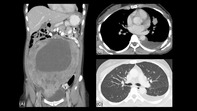

## INTRODUCTION

Krukenberg tumour is a metastatic ovarian cancer characterized by mucin‐rich signet‐ring adenocarcinoma.^1^ Such cancer usually develops from the gastrointestinal tract and, less frequently, from other locations such as breast, gallbladder, thyroid and kidney.^2^ The incidence of Krukenberg tumours is higher in Asian countries (20%) compared with the western population (4%). It is postulated that the high prevalence of gastric cancer accounts for its predominance.^3^ Krukenberg tumours only account for less than 2% of all ovarian carcinoma, therefore data on lung cancer with ovarian metastases is even more sparse.^4^ We report a rare presentation of lung adenocarcinoma with ovarian metastasis with particular importance to its management decision.

## CASE REPORT

A 38‐year‐old woman presented with a 2‐year history of progressive abdominal distension. She noticed abdominal fullness the size of a tennis ball, which subsequently progressed to the size of a soccer ball. She has no known medical illness. The patient has never smoked and has no history of exposure to secondhand smoke. She has been a full‐time housewife with no history of occupational exposure to carcinogens. She is nulliparous with a regular 28‐day cycle of a menstrual period.

Abdominal examination revealed a huge immobile suprapubic mass up to 30 weeks in size. Chest examination demonstrated reduced air entry and vocal resonance over the left lower zone chest wall with stony dullness upon percussion. Breast examination was normal with no palpable breast mass. There was no palpable lymphadenopathy and other systemic review was unremarkable.

**FIGURE 1:**
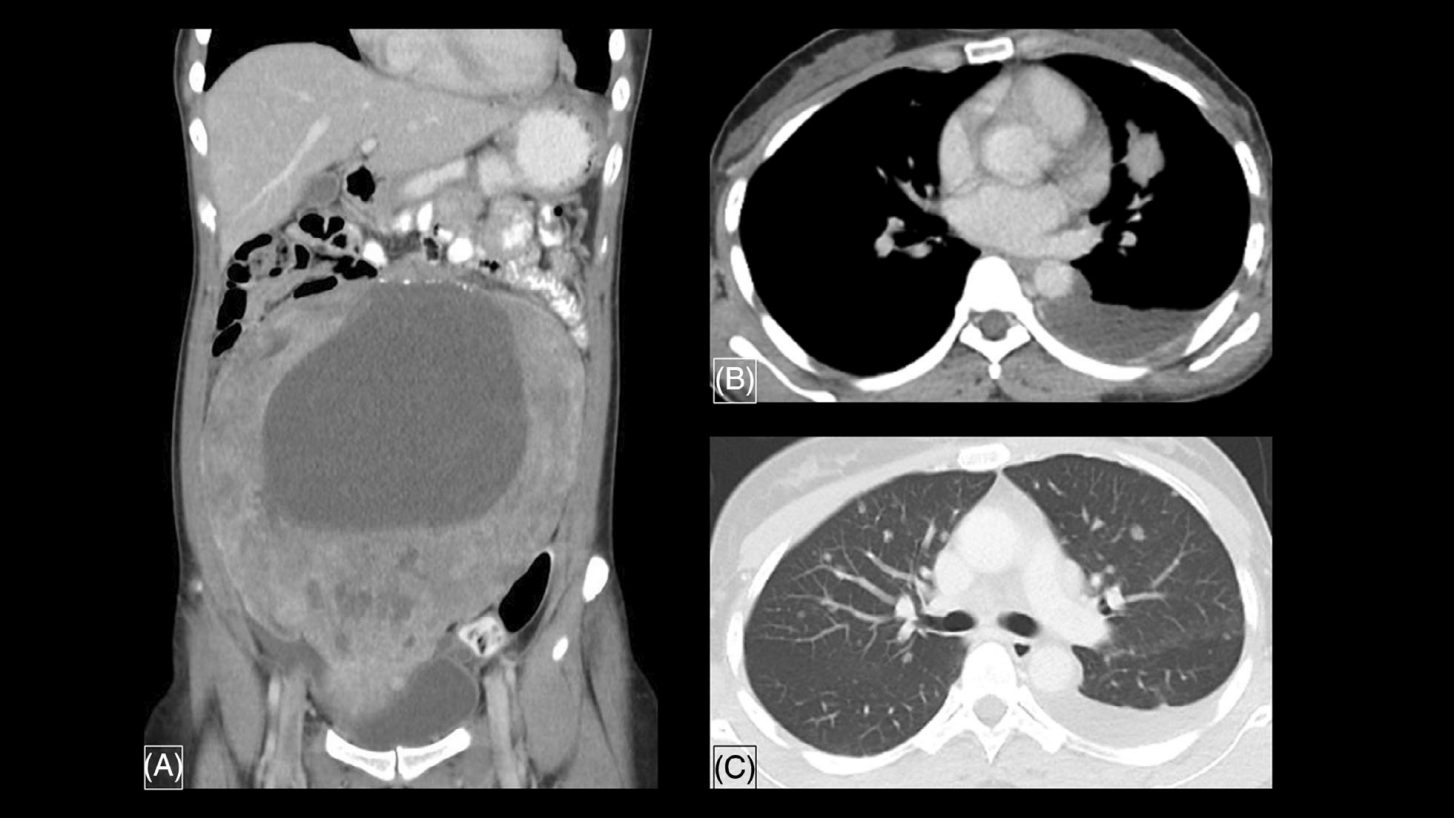
A contrast‐enhanced CT scan of the abdomen showing a large solid cystic mass arising from the pelvis occupying almost the entire lower abdomen, minimal free fluid is seen. (A) A solid irregular lung lesion is seen at the left upper lobe (B). HPE proven lung adenocarcinoma with ipsilateral pleural effusion with multiple bilateral small lung metastases (C)

Gynaecological transabdominal ultrasonography identified a huge ovarian mass with a size of 20 × 20 cm (anteroposterior × transverse diameter). Computed tomography (CT) of thorax, abdomen and pelvis depicts a large solid‐cystic pelvic mass, with bilateral ovarian cysts and left hydrosalpinx. There is a solid irregular lung lesion is seen at the left upper lobe, ipsilateral pleural effusion and multiple bilateral small lung metastases (Figure 1).

**FIGURE 2:**
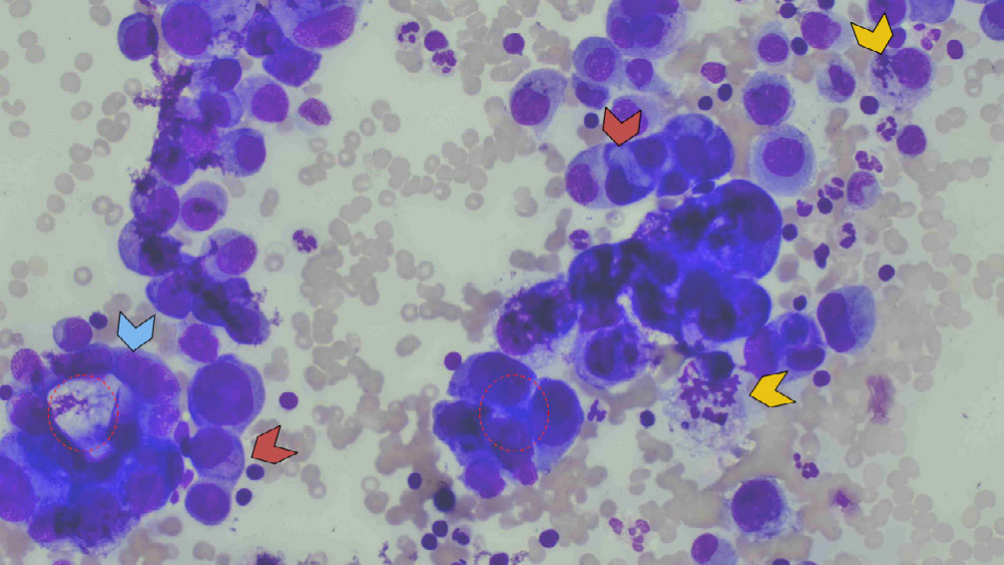
Papanicolou smear in pleural fluid cytology reviewed the presence of adenocarcinoma in acinar pattern (arrowhead blue). These neoplastic cells exhibit pleomorphic nuclei with cytoplasmic vacuolation (arrowhead orange). Mitosis is easily seen (arrowhead yellow)

**FIGURE 3:**
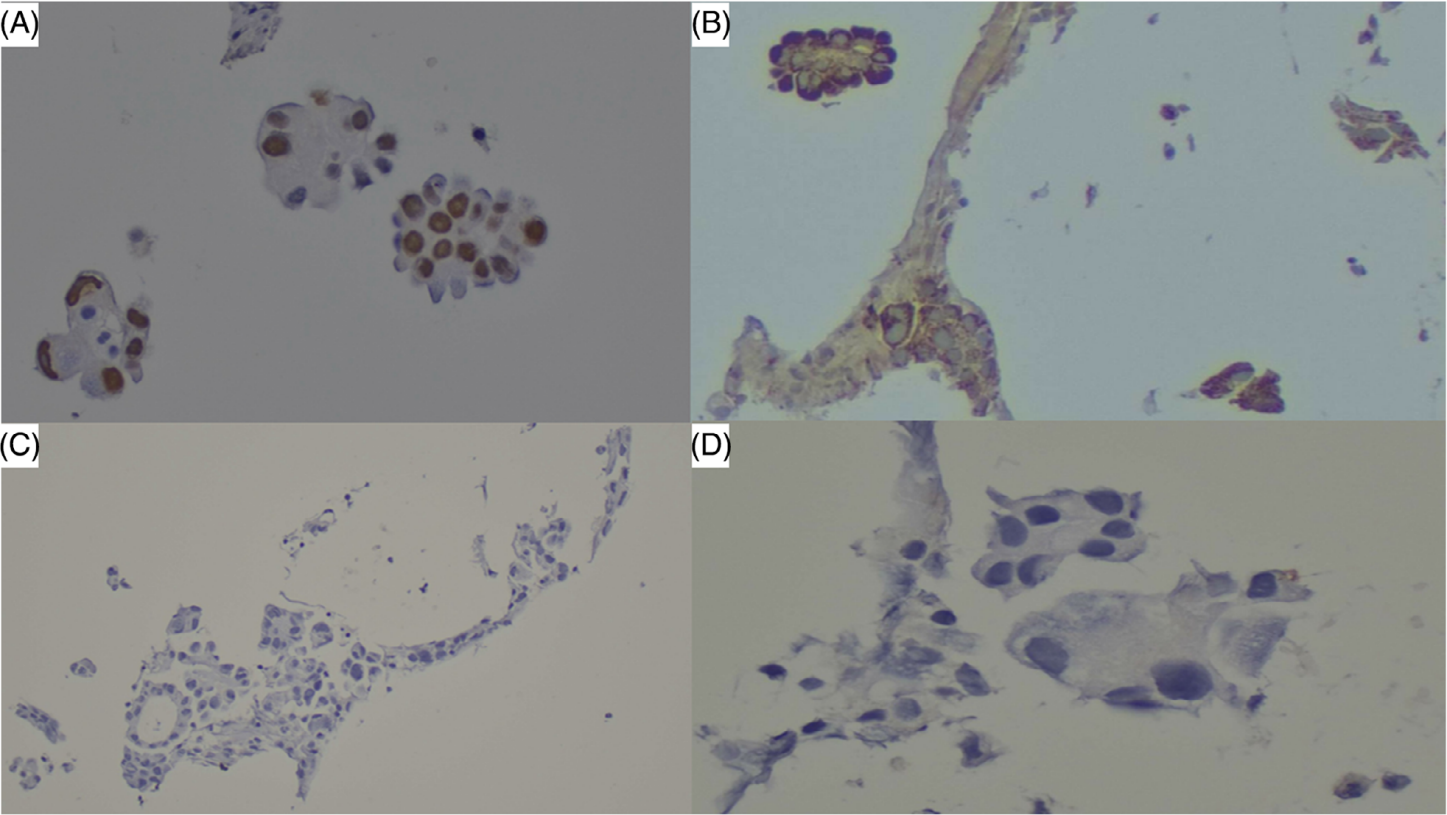
Expression of TTF‐1 as a brown nuclear stain (A) and Napsin A as a purple cytoplasmic stain (B) was identified in tumour cells. Immunohistochemical staining for Oestrogen receptor (ER) and Progesterone receptor (PR) were negative (C and D, respectively)

The patient underwent several procedures to identify the primary source of malignancy. Pipelle tissue sampling was negative for malignancy, and the visualized colon was normal during colonoscopy. Left pleural tapping was performed and cytology showed the presence of neoplastic cells with acinar pattern suggestive of adenocarcinoma (Figure 2). The immunohistochemistry results were positive for thyroid transcription factor‐1 (TTF‐1) and Napsin A, and negative for CK20, PAX8, oestrogen receptor (ER) and progesterone receptor (PR) markers (Figure 3). Discussion during tumour board meeting concluded that the cytomorphology and immunohistochemistry profile is suggestive of adenocarcinoma of lung primary with ovarian metastasis, with TNM staging T4N2M1c (stage Ivb).

Further molecular testing for lung adenocarcinoma oncogenic mutation was negative for epidermal growth factor receptor (EGFR), Anaplastic lymphoma kinase (ALK) c‐ros oncogene 1, (ROS‐1) and Programmed cell death ligand 1 (PD‐L1). The molecular testing for tumour sample is done via the AmoyDx Pan Lung cancel panel, which is a real‐time PCR assay for qualitative detection of alterations in EGFR, ALK and ROS‐1. The VENTANA PD‐L1 Assay is used to assess PD‐L1 levels with a the qualitative immunohistochemical assay via monoclonal anti‐PD‐L1 clone SP263. To date, she is currently on doublet platinum chemotherapy (cisplatin and pemetrexed), which shows partial response with a significant reduction of ovarian tumour size from 20 × 20 cm to 8 × 10 cm.

## DISCUSSION

The exact mechanism of Krukenberg tumour metastasis is still unknown, with three proposed mechanisms including tumour spread via lymphatic system, hematogenous system, and transcoelomic pathway.^5^ The risk of metastasis is higher with an increased number of positive metastatic lymph nodes, which is seen in our case with mediastinal and para‐aortic lymphadenopathy.^5^ Apart from the common symptoms of abdominal pain and distension, Krukenberg tumours can stimulate ovarian stroma and provoke hormone production, resulting in vaginal bleeding, menstrual changes, hirsutism and virilization.^6^

The diagnostic challenge, in this case, is to identify the origin of adenocarcinoma. Adenocarcinomas are malignant neoplasms arising from epithelial tissue of the glands. The histology includes the identification of pleomorphic nucleoli with a glandular structure under light microscopy.^7^ It can arise from multiple sites of the body, including breast, lung gastrointestinal tract and prostate.^7^ Immunohistochemistry is an important tool to identify the origin of adenocarcinoma. Positive staining for Thyroid transcription factor‐1 (TTF‐1P) and Napsin A in our case is strongly suggestive of adenocarcinoma of the lung.^8^ By combining napsin A and TTF‐1, the sensitivity and specificity for primary lung adenocarcinoma are 91.0% and 93.8%, respectively.^8^ A panel of immunohistochemical stains including PAX8, ER/PR are negative in our case, which carries great value to exclude female genital tracts and breast adenocarcinoma.^9^ Based on the patient's non‐smoker status, female gender, and Asian ethnicity, we suspect the presence of other oncogene mutations in addition to those already tested for, such as Kirsten rat sarcoma viral oncogene homologue (KRAS), B‐type Rat Kinase (BRAF) rearrangements during transfection (RET), neurotrophic receptor tyrosine kinase (NTRK), human epidermal growth factor receptor 2 (HER2). If feasible, we recommended additional testing to inform treatment decisions. However, given the unavailability of testing for rarer oncogenic mutations in our center and the patient's inability to afford further molecular testing, we have opted to proceed with chemotherapy as the primary treatment strategy.

Krukenberg tumour, although rare, should be considered as a differential diagnosis during the investigation of ovarian/pelvic mass as this condition poses a different route of investigation, treatment and prognosis. Traditionally, Krukenberg tumour is diagnosed after oophorectomy or total abdominal hysterectomy with bilateral salpingo‐oophorectomy. In contrast, in our patient's journey to diagnosis, extensive surgical intervention to obtain sample for definitive tissue diagnosis was avoided as there was a less invasive source to be sampled, which is the left pleural effusion. Although we lack definitive histological proof of ovarian metastasis, the patient's cytomorphology and immunohistochemistry profile suggest adenocarcinoma of lung primary with ovarian metastasis. Our tumour board meeting agreed that the typical moth‐eaten cysts appearance of the ovary supports this diagnosis. To date, the patient has shown a significant reduction in ovarian tumour size in response to chemotherapy, indicating good treatment response without extensive surgical intervention.

Since the Krukenberg tumour is a metastatic disease from other organs, it is classified as a stage IV disease, that carries a poor prognosis.^10^ The management of Krukenberg is still controversial. Some studies suggested that cytoreductive surgery incorporating resection of ovarian metastases might prolong survival in gastric carcinoma.^11^ On the other hand, a retrograde analysis of 133 patients did not recommend patients to undergo ovarian metastasectomy if the primary stomach lesion had not or could not be resected, or ascites were detected.^12^ Post‐operative complications such as surgical site infection and intraabdominal collection also carry a risk for patients who underwent chemotherapy.^11^ To date, there are no studies regarding the efficacy of surgical debulking of Krukenberg tumour originating from lung adenocarcinoma due to the paucity of data. Although surgical resection of ovarian metastases remains a controversial option, our patient's experience highlights the potential benefits of chemotherapy in advanced lung cancer. Nevertheless, surgical options would be a priority if complications occur including ovarian torsion, tumoral bleed or local compression.^13^

In conclusion, early identification of the primary source of the Krukenberg tumour is paramount to avoid invasive diagnostic surgical interventions for ovarian metastasis. Furthermore, adequate IHC staining is useful in confirming the origin of metastatic adenocarcinoma.

## AUTHOR CONTRIBUTIONS

All listed authors contributed to the article.

## CONFLICT OF INTEREST STATEMENT

None declared.

## ETHICS STATEMENT

The authors declare that appropriate written informed consent was obtained for the publication of this manuscript and accompanying images.

## Data Availability

The data that support the findings of this study are available from the corresponding author upon reasonable request.

## References

[1] Yakushiji M , Tazaki T , Nishimura H , Kato T . Krukenberg tumors of the ovary: a clinicopathologic analysis of 112 cases. Nihon Sanka Fujinka Gakkai Zasshi. 1987;39(3):479–85. 3031182

[2] Jeung YJ , Ok HJ , Kim WG , Kim SH , Lee TH . Krukenberg tumors of gastric origin versus colorectal origin. Obstetr Gynecol Sci. 2015;58(1):32–9. 10.5468/ogs.2015.58.1.32 PMC4303750 25629016

[3] Moore RG , Chung M , Granai CO , Gajewski W , Steinhoff MM . Incidence of metastasis to the ovaries from nongenital tract primary tumors. Gynecol Oncol. 2004;93(1):87–91. 15047218 10.1016/j.ygyno.2003.12.039

[4] Kiyokawa T , Young RH , Scully RE . Krukenberg tumors of the ovary: a clinicopathologic analysis of 120 cases with emphasis on their variable pathologic manifestations. Am J Surg Pathol. 2006;30(3):277–99. 16538048 10.1097/01.pas.0000190787.85024.cb

[5] Aziz M , Kasi A . Krukenberg tumor. StatPearls [Internet]. Treasure Island: StatPearls Publishing; 2021. 29489206

[6] Csömör S , Melczer Z , Kazy Z . Data to the clinical manifestation of the Krukenberg tumour. Acta Chir Hung. 1998;37(1–2):101–6. 10196618

[7] Noguchi M , Morikawa A , Kawasaki M , Matsuno Y , Yamada T , Hirohashi S , et al. Small adenocarcinoma of the lung histologic characteristics and prognosis. Cancer. 1995;75(12):2844–52. 7773933 10.1002/1097-0142(19950615)75:12<2844::aid-cncr2820751209>3.0.co;2-#

[8] Kadivar M , Boozari B . Applications and limitations of immunohistochemical expression of “Napsin‐A” in distinguishing lung adenocarcinoma from adenocarcinomas of other organs. Appl Immunohistochem Mol Morphol. 2013;21(3):191–5. 22914608 10.1097/PAI.0b013e3182612643

[9] Lerwill MF . Current practical applications of diagnostic immunohistochemistry in breast pathology. Am J Surg Pathol. 2004;28(8):1076–91. 15252316 10.1097/01.pas.0000126780.10029.f0

[10] Gilliland R , Gill PJ . Incidence and prognosis of Krukenberg tumour in Northern Ireland. Br J Surg. 1992;79(12):1364–6. 1336701 10.1002/bjs.1800791241

[11] Seow‐En I , Hwarng G , Tan GH , Ho LM , Teo MC . Palliative surgery for Krukenberg tumors–12‐year experience and review of the literature. World J Clin Oncol. 2018;9(1):13–9. 29468133 10.5306/wjco.v9.i1.13 PMC5807888

[12] Peng W , Hua RX , Jiang R , Ren C , Jia YN , Li J , et al. Surgical treatment for patients with Krukenberg tumor of stomach origin: clinical outcome and prognostic factors analysis. PLoS One. 2013 Jul 9;8(7):e68227. 23874550 10.1371/journal.pone.0068227 PMC3706522

[13] Bibi S , Memon S , Qazi RA . Acute abdomen secondary to torsion of Krukenberg tumour. JPMA J Pak Med Assoc. 2011;61(8):819. 22356011

